# Association between milk pump type and free fatty acid concentrations on dairy farms with automated milking systems

**DOI:** 10.3168/jdsc.2024-0666

**Published:** 2025-01-10

**Authors:** Hannah M. Woodhouse, Stephen J. LeBlanc, Trevor J. DeVries, Karen J. Hand, David F. Kelton

**Affiliations:** 1 Department of Population Medicine, University of Guelph, Guelph, Ontario, Canada N1G2W1; 2 Department of Animal Biosciences, University of Guelph, Guelph, Ontario, Canada N1G2W1; 3 Precision Strategic Solutions, Puslinch, Ontario, Canada N0B2J0

## Abstract

High levels of free fatty acids (FFA) in milk (≥1.20 mmol FFA/100 g of fat) indicate excessive milk fat breakdown and compromise milk quality. Automated milking systems (AMS) have become more common in the dairy industry, but questions about their effect on milk quality, including FFA, remain. On average, AMS-milked herds have greater FFA levels in bulk tank milk than parlor-milked herds. The difference in milk pump type between some AMS and parlor systems may be a contributing factor. The objective of this study was to investigate whether a positive displacement milk pump (PDMP) would be associated with greater FFA when compared with a centrifugal milk pump (CMP) on AMS farms. We hypothesized that a PDMP would be associated with greater FFA levels because of the potential impact of high flow rates on milk fat globules. We conducted an observational pilot study using farm and milk quality data collected from Ontario, Canada, AMS herd visits. Monthly average milk composition data surrounding the farm visit date were obtained from the Dairy Farmers of Ontario and included FFA concentration, milk fat (% weighted volume), milk protein (% weighted volume), and milk shipment volume (L). A linear regression analysis was conducted with monthly average FFA as the outcome and pump type as the explanatory variable of interest, with other factors identified in previous research to be associated with increased FFA accounted for in the model. One hundred twenty-one AMS herds were visited between 2019 and 2021, with an average (± SD) monthly FFA of 0.86 ± 0.18 mmol/100 g of fat. Seventy-four farms (61%) had a PDMP, and the average FFA level was 0.88 mmol FFA/100 g of fat, which was above the provincial industry average and greater than AMS farms with a CMP. The results suggest that FFA may be slightly greater on AMS farms with PDMP (β = 0.04 mmol FFA/100 g milk fat, 95% CI −0.02 to 0.10), but the difference was not statistically significant and is small compared with other previously identified FFA factors. However, this could be due to a small sample size and few study farms with high FFA levels.

## Highlights

• Milk harvested from AMS is associated with greater FFA than parlors.
• A distinguishable difference between some AMS and parlors is the milk pump type.
• Some AMS have a PDMP rather than a CMP.
• PDMP type and increased FFA were weakly correlated.
• There are more influential factors for increased FFA in AMS herds than milk pump type.

## Graphical Abstract

**Figure:**
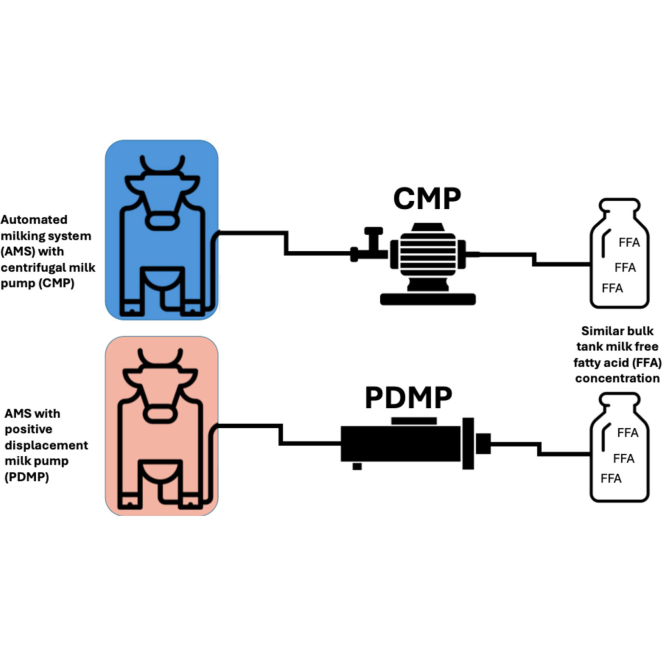
**Summary:** The prevalence of automated milking systems (AMS) is increasing in the dairy industry, as are free fatty acids (FFA). High levels of FFA in bulk tank raw milk threaten milk quality and are more common in AMS herds than parlor-milked herds. A positive displacement milk pump (PDMP) rather than a centrifugal milk pump (CMP) in some AMS may be a contributing factor. This pilot study aimed to investigate whether a PDMP was associated with increased FFA. Descriptive statistics and a linear regression analysis were conducted using 121 Ontario, Canada, AMS herd data to compare pump type and FFA concentration. Seventy-four farms (61%) had a PDMP, and the average FFA level was 0.88 mmol FFA/100 g of fat, which was above the provincial industry average and greater than AMS farms with a CMP. However, the linear regression analysis detected a weak correlation between pump type and FFA concentration, suggesting that other AMS factors are more influential.

Some associations have been reported between milk harvested using an automated milking system (**AMS**) and increased SCC, bacteria levels, or freezing point (Klungel et al., 2000; de Koning et al., 2003; Cogato et al., 2021). Recently, the concentration of free fatty acids (**FFA**) has been added to these measures of milk quality in some countries, including Canada. Above concentrations of 1.20 mmol FFA/100 g of fat (Wiking et al., 2017), FFA levels can impair milk quality in terms of milk structure, function, and taste (Deeth, 2006; Skeie, 2007; Kamath et al., 2008). Milk fat breakdown (referred to as lipolysis) results in FFA and can be caused by milk fat globule membrane damage that exposes triacylglycerol (**TAG**) molecules to lipolytic enzymes (Deeth, 2006). Various forms of lipolysis can result in FFA, and induced lipolysis refers to the milk fat globule membrane damage that results primarily from factors related to milk handling and transport (Evers and Palfreyman, 2001; Rasmussen et al., 2006; Wiking et al., 2019).

Automated milking systems may be at greater risk of increased FFA compared with parlor-milked herds due to the increased occurrence of induced lipolysis (Rasmussen et al., 2006; Wiking et al., 2006, 2019). Potential air leakages from quarter milking and higher levels of air admission to transport milk through longer, narrow pipelines on AMS farms have been reported as risk factors for increased FFA (Rasmussen et al., 2006; Wiking et al., 2006, 2019). Temperature fluctuations and milk freezing in the bulk tank are common risk factors for elevated FFA on AMS farms because of smaller volumes of milk entering the bulk tank during the initial filling, or the bulk tank agitator being turned on too soon (de Koning et al., 2003; Rasmussen et al., 2006; Wiking et al., 2019). Higher milking frequencies (i.e., >2×/d, a characteristic of AMS herds) can also increase lipolysis and FFA due to insufficient milk fat globule membrane synthesis caused by shortened milking intervals (de Koning et al., 2003; Wiking et al., 2006, 2019).

There are additional factors associated with increased FFA that are not specific to AMS. Previous research on conventional farms in Ontario, Canada, reported lower FFA concentrations than on organic or grass-fed farms (Woodhouse et al., 2024a). When looking at Ontario conventional herds exclusively, there was an association between lower milk protein percentage and elevated FFA, likely due to a weakened milk fat globule membrane structure, which protein plays an important role in (Woodhouse, 2024). There was also an association between increased milk fat production allotments (referred to as incentive days where producers can ship milk beyond their allotted quota) and elevated FFA, possibly due to increased fat supplementation use and herd demographic changes during these times (Woodhouse et al., 2024a; Woodhouse, 2024). Incentive days normally occur in the fall months to meet production demands, and this association helped to explain seasonal variation in FFA levels (Evers and Palfreyman, 2001; Woodhouse, 2024). Herd size is also a reported FFA risk factor, with the dilution effect of lower FFA in larger herds (Oleggini et al., 2001; Wiking et al., 2019).

The milk pump type is one of the understudied potential factors that could influence FFA concentrations (Wiking et al., 2003, 2005; Rasmussen et al., 2006). Milk traveling from the milking machine to the bulk tank cannot rely on gravity flow due to pipeline length, turns, and elevated sections (Spencer, 2011). A milk pump, along with air admission (vacuum), functions to provide enough force to help milk travel (Spencer, 2011). Most of the milk pumps used on farms are centrifugal milk pumps (**CMP**), which have a spinning impeller that rotates to accelerate milk outwards and through the pipelines (Spencer, 2011; Tetra Pak, 2015). Positive displacement milk pumps (**PDMP**; also referred to as bladder pumps) suction milk into a cavity, then a cylindrical silicone tube forces milk out into the pipelines with higher pressure (Srivastava, 2024). Most CMP have variable speed, meaning that they do not run constantly (Tetra Pak, 2015; Srivastava, 2024). A CMP can also slow down the speed of milk exiting the pump, therefore reducing force and milk stress (Tetra Pak, 2015; Srivastava, 2024). A PDMP, however, has a constant flow rate with higher pressure, which has the potential to damage the milk fat globule membrane (Srivastava, 2024).

Limited research exists on the effects of pumping milk on FFA concentrations in bulk tank milk. To our knowledge, no studies have explored the association of pump type with FFA concentration. Therefore, the objective of this pilot study was to investigate the relationship between pump type and FFA concentration in bulk tank milk on AMS farms. We hypothesized that greater FFA levels would be observed on farms with a PDMP.

This research was a pilot observational study using data collected in 2 previous studies that recruited Ontario dairy farms with AMS (Matson et al., 2021; Woodhouse et al., 2024b) between July 10, 2019, and June 30, 2021. No a priori sample size was calculated for the present analysis because the number of farms with appropriate data was limited to those available in the underlying studies. These AMS farms received a one-time visit, including the administration of a questionnaire about farm demographics and herd management (Matson et al., 2021; Woodhouse et al., 2024b). Written permission was received to use the data. No animal was handled as part of these studies, so an animal care utilization protocol was not required. These studies were approved by the Research Ethics Board (REB) of the University of Guelph REB no. 22-07-031 (Woodhouse et al., 2024b) and REB no. 19-01-012 (Matson et al., 2021). Data for this pilot project were analyzed using commercial statistical software (STATA version 16.0, StataCorp).

From the previous studies' datasets, the visit date, number of AMS, pump type, and monthly average milk components (FFA [mmol/100 g of fat], fat [% wt/vol], protein [% wt/vol], and milk shipment yield [L]) of the visit date for each AMS herd were extracted and merged into a single dataset. Previous research (Woodhouse, 2024) did not detect an association between FFA and SCC, so the analyses did not include bulk tank SCC. Bacterial counts were also excluded due to minimal reporting of this milk quality parameter compared with the other milk components. Researchers were not blinded to the study data. Any AMS herds with more than one pump type (i.e., different AMS models) were excluded. Any herds that were organic or grass-fed were also removed from the dataset to control for the potential confounding effects of farm type and to enable us to account for the association between FFA and incentive days in the model (different for organic and grass-fed herds; Woodhouse, 2024). Only herds with every-other-day milk pick-up were included to account for milk volume in the model.

A linear regression model was constructed with monthly average FFA as the outcome variable of interest. The explanatory variable of interest was pump type (CMP or PDMP). Based on previous literature (Wiking et al., 2019; Woodhouse, 2024; Woodhouse et al., 2024b), other explanatory variables were added to the model as fixed effects. We (Woodhouse, 2024) previously identified an association between incentive days and increased odds of elevated FFA, so the number of incentive days for the month of the farm visit was included in the analysis. The average monthly milk protein percentage was also included because previous studies have detected an association with FFA (Thomson et al., 2005; Hanuš et al., 2008; Marcondes et al., 2014; Woodhouse, 2024). The model did not include average milk fat percentage due to its intervening effect on incentive days and high correlation with average milk protein. Herd size may also influence FFA (Wiking et al., 2019), so this analysis used the number of AMS units per farm to represent herd size. All continuous variables of interest were assessed for linearity using locally weighted scatterplot smoothing. The number of incentive days was categorized into <3 and 3 incentive days in the corresponding visit month because we previously observed an association between 3 incentive days and elevated FFA (Woodhouse, 2024). The number of AMS units per farm was not linear and therefore categorized into 1, 2, or ≥3 robots.

Data from 121 AMS farms were available for this study; 66 farms came from Matson et al. (2021) and 55 from Woodhouse et al. (2024b). The frequency distributions of the AMS farm data are reported in Table 1. There were 74 farms (61%) with a PDMP and 47 farms (39%) with a CMP. The largest herd had 11 AMS units, whereas 33 farms (27%) had a single AMS unit. Seventy-five percent (n = 3) of the farms with an elevated FFA (≥1.2 mmol/100 g of fat) had a PDMP. Descriptive statistics of average monthly milk components included in the analyses are reported in Table 2. Farms with a PDMP had higher maximum monthly average FFA, fat, protein, and milk shipment volumes than farms with a CMP.

**Table 1:** Frequency distribution (n [%] of category within pump type) of data from farms with automated milking systems (AMS) by milk pump type: centrifugal milk pump (CMP) or positive displacement milk pump (PDMP)

Variable	Total (n = 121)	Farms with CMP (n = 47)	Farms with PDMP (n = 74)
Elevated FFA (≥1.20 mmol/100 g of fat)			
No	117 (96.7)	46 (97.9)	71 (96.0)
Yes	4 (3.3)	1 (2.1)	3 (4.1)
Number of AMS			
1	33 (27.3)	9 (19.2)	24 (32.4)
2	59 (48.7)	23 (48.9)	36 (48.7)
≥3	29 (24.0)	15 (31.9)	14 (18.9)
3 quota incentive days in the month of data collection^1^			
No	89 (73.6)	55 (44.3)	34 (72.3)
Yes	32 (26.4)	19 (25.7)	13 (27.7)

1 Incentive days are monthly increases in producer quota allotment to meet production demands (up to 3 d of extra milk production).

**Table 2:** Descriptive statistics of average monthly milk components for the month of farm visit for the collection of data on farms with automated milking systems with a centrifugal milk pump (CMP, n = 47) or positive displacement milk pump (PDMP, n = 74)

Milk component	Mean (± SD)		Median		Minimum		Maximum	
	CMP	PDMP	CMP	PDMP	CMP	PDMP	CMP	PDMP
Monthly average FFA (mmol/100 g of fat)	0.83 ± 0.18	0.88 ± 0.18	0.86	0.88	0.44	0.45	1.26	1.49
Monthly average fat (% weight by volume)	4.0 ± 0.2	4.0 ± 0.3	3.9	4.0	3.6	3.5	5.0	5.2
Monthly average protein (% weight by volume)	3.1 ± 0.1	3.1 ± 0.2	3.1	3.1	2.9	2.9	3.7	3.8
Monthly average milk shipment volume at each pick-up (L)	7,342 ± 4,622	6,151 ± 3,786	6,050	5,044	2,381	2,196	24,715	24,916

Table 2, Table 3 report the average monthly FFA concentration for farms with a PDMP (0.88 mmol/100 g of fat) being greater than farms with a CMP (0.83 mmol/100 g of fat). However, when accounting for other variables, the CI around the estimated FFA difference was mostly <0 (*P* = 0.19). Higher average protein was associated with lower FFA (β = −0.04, *P* < 0.01), which aligns with previous study results (Thomson et al., 2005; Marcondes et al., 2014; Woodhouse, 2024). The presence of 3 incentive days in the month of milk collection was associated with higher FFA the model (β = 0.06, *P* = 0.08) compared to <3 incentive days. This result also aligns with previous research findings (Woodhouse, 2024).

**Table 3:** Results of a multivariable linear regression model of the association of automated milking system (AMS) milk pump type with monthly average free fatty acid concentration

Variable	β-Coefficient	95% CI	*P-*value
Pump type			
CMP	Referent		
PDMP	0.04	−0.02, 0.10	0.19
Number of AMS			
1	Referent		
2	0.01	−0.07, 0.08	0.88
≥3	−0.05	−0.14, 0.04	0.25
3 incentive days^1^			
No	Referent		
Yes	0.06	−0.01, 0.13	0.08
Average protein (% weight by volume: per 1% increase)	−0.04	−0.06, −0.02	<0.01

1 Incentive days are monthly increases in producer quota allotment to meet production demands (up to 3 d of extra milk production).

The inability to detect a statistically significant FFA difference between pump types could be due to a small sample size and few farms with higher FFA levels. Given that this is the first study investigating pump type as a potential contributor to FFA in bulk tank milk, there were no data on which to base a sample size calculation. Although a larger study would provide more power, the magnitude of the effect suggested by the present analysis indicates that it may not be large enough to be practically important. We suspect that other factors, such as increased average milking frequency in AMS herds, are more influential on FFA levels (de Koning et al., 2003; Wiking et al., 2006, 2019). Future studies could include milking frequency as a fixed effect in the analysis if a larger dataset with more frequent FFA test values were used. This study did not include average milking frequency for the month around data collection because the data were not captured across the 2 original datasets.

In conclusion, this exploratory pilot study on farms with AMS did not detect a significant association between milk pump type and the monthly average concentration of FFA in bulk tank milk. This suggests that other factors may be more influential on FFA levels on AMS farms. However, we suggest that pump type should be included with other variables in future studies that are investigating AMS factors associated with milk FFA levels.
